# Exercise‐heat stress with and without water replacement alters brain structures and impairs visuomotor performance

**DOI:** 10.14814/phy2.13805

**Published:** 2018-08-22

**Authors:** Matthew T. Wittbrodt, Michael N. Sawka, J. C. Mizelle, Lewis A. Wheaton, Melinda L. Millard‐Stafford

**Affiliations:** ^1^ School of Biological Sciences Georgia Institute of Technology Atlanta Georgia; ^2^ Department of Kinesiology East Carolina University Greenville North Carolina

**Keywords:** Brain anatomy, cognition, dehydration, heat stress, motor function

## Abstract

Effects of exercise‐heat stress with and without water replacement on brain structure and visuomotor performance were examined. Thirteen healthy adults (23.6 ± 4.2 years) completed counterbalanced 150 min trials of exercise‐heat stress (45°C, 15% RH) with water replacement (EHS) or without (~3% body mass loss; EHS‐DEH) compared to seated rest (CON). Anatomical scans and fMRI Blood‐Oxygen‐Level‐Dependent responses during a visuomotor pacing task were evaluated. Accuracy decreased (*P* < 0.05) despite water replacement during EHS (−8.2 ± 6.8% vs. CON) but further degraded with EHS‐DEH (−8.3 ± 6.4% vs. EHS and −16.5 ± 10.2% vs. CON). Relative to CON, EHS elicited opposing volumetric changes (*P* < 0.05) in brain ventricles (−5.3 ± 1.7%) and periventricular structures (cerebellum: 1.5 ± 0.8%) compared to EHS‐DEH (ventricles: 6.8 ± 3.4, cerebellum: −0.7 ± 0.7; thalamus: −2.7 ± 1.3%). Changes in plasma osmolality (EHS: −3.0 ± 2.1; EHS‐DEH: 9.3 ± 2.1 mOsm/kg) were related (*P* < 0.05) to thalamus (*r* = −0.45) and cerebellum volume (*r* = −0.61) which, in turn, were related (*P* < 0.05) to lateral (*r* = −0.41) and fourth ventricle volume (*r* = −0.67) changes, respectively; but, there were no associations (*P* > 0.50) between structural changes and visuomotor accuracy. EHS‐DEH increased neural activation (*P* < 0.05) within motor and visual areas versus EHS and CON. Brain structural changes are related to bidirectional plasma osmolality perturbations resulting from exercise‐heat stress (with and without water replacement), but do not explain visuomotor impairments. Negative impacts of exercise‐heat stress on visuomotor tasks are further exacerbated by dehydration.

## Introduction

Prevention of significant dehydration by replacing fluids during exercise in the heat attenuates adverse physiological effects such as reduced aerobic performance (Kenefick 2018). Since dehydration is a common stressor occurring with prolonged heat exposure in various occupational and military settings, whether fluid replacement mitigates cognitive‐motor deficits is also an important question. Despite inconclusive scientific evidence (IOM, 2004; Cheuvront and Kenefick 2014), it is commonly suggested that dehydration adversely impacts cognitive‐motor and central nervous system function. However, conflicting findings exist regarding the impact of dehydration on cognitive‐motor performance, with some studies observing marked impairments (Sharma et al. 1986; Gopinathan et al. 1988; Cian et al. 2000; Baker et al. 2007a; Watson et al. 2015), others no differences (Hogervorst et al. 1996; Szinnai et al. 2005; Adam et al. 2008; Armstrong et al. 2012; Ely et al. 2013; Wittbrodt et al. 2015b; van den Heuvel et al. 2017), and even one with improved performance (Bandelow et al. 2010). No singular explanation accounts for these disparate results; but, explanations likely include inconsistencies within the assessed cognitive‐motor domains coupled with study design considerations such as the magnitude of body water deficit and the method used to induce dehydration (i.e., sweat loss due to exercise and/ or heat stress).

Previous studies examining the effects of dehydration on cognitive‐motor function typically assess performance with computerized tasks utilizing a higher‐order cognitive domain (e.g., executive control, information processing, memory) that require a motor response (e.g., button press). While these assessments hold value, they may assess overly broad cognitive‐motor functions. Tasks isolating visuomotor responses allow researchers to examine the influence of a cognitive system (vision) on motor function through networks involving visual cortex, sensorimotor areas, parietal cortex, and basal ganglia (Fitts 1954; Cisek and Kalaska 2010). Some studies have suggested motor coordination may be impaired following dehydration (Cian et al. 2000); however, a primary component of visuomotor functioning, the ability to accurately process temporal information (Buhusi and Meck 2005), has not been previously examined. Adequate visuomotor performance is essential to human‐system interactions, and dysfunction (potentially from deficient visuomotor timing) might explain errant performance in tasks such as driving proficiency (Watson et al. 2015), pilot simulations (Lindseth et al. 2013), and sporting skills (Baker et al. 2007b; Smith et al. 2012) are degraded following dehydration.

Controversy also exists whether dehydration alters brain water content and anatomical structures. Early animal autopsy studies observed no changes in brain volume following severe dehydration (−10% of body mass, BM) (Falck and Scheffer 1854; Nose et al. 1983) suggesting homeostatic neural mechanisms adequately offset large perturbations to body water balance (Gullans and Verbalis 1993; De Petris et al. 2001). Subsequent human in vivo neuroimaging studies usually confirm total brain volume is not altered with dehydration (Dickson et al. 2005; Kempton et al. 2009; Watson et al. 2010; Nakamura et al. 2014; Meyers et al. 2016); however, lateral ventricle volume is observed to either expand (Kempton et al. 2009, 2011a), shrink (Watson et al. 2010), or not change (Dickson et al. 2005; Streitburger et al. 2012; Meyers et al. 2016). Lateral ventricle expansion is also associated with cognitive‐motor decrements during aging and/or neurological disturbances and likely attributed to adjacent periventricular gray matter atrophy (Johnstone et al. 1976; Coffey et al. 1992; Breteler et al. 1994). To date, only one study examined white and gray matter following dehydration, finding no volumetric change with ~2% BM loss achieved with fluid restriction (Streitburger et al. 2012). Furthermore, the other previous magnetic resonance imaging (MRI) studies employing fluid restriction or exercise‐heat stress protocols have induced nominal dehydration (<2% BM loss) (Kempton et al. 2011a; Meyers et al. 2016) or have not controlled for the effects of prior exercise‐heat stress using only a preexercise rest condition (Kempton et al. 2009; Watson et al. 2010) or an exercise protocol with less thermal stress (Kempton et al. 2011a). Therefore, the known impact of dehydration as a result of exercise‐heat stress on brain structures remains elusive.

Thus, a comprehensive study is needed to examine: (1) whether dehydration due to exercise‐heat stress alters brain structures and impairs fundamental visuomotor performance, and (2) if prevention of dehydration via water replacement during exercise‐heat stress attenuates structural and functional changes. Given the prevalence of studies employing the exercise‐heat stress method for dehydration and variability of findings reporting either impairments (Sharma et al. 1986; Gopinathan et al. 1988; Baker et al. 2007a; Tomporowski et al. 2007) or no effect (Serwah and Marino 2006; Morley et al. 2012; Ely et al. 2013) on performance across multiple cognitive‐motor domains, we utilized this method relevant across multiple scenarios (military, athletic, occupational) and, specifically, at a level of dehydration known to elicit homeostatic responses (Cheuvront et al. 2013). Based upon our recent meta‐analysis (Wittbrodt and Millard‐Stafford 2018), we hypothesized dehydration would impair performance during a prolonged visuomotor task, but water replacement during exercise‐heat stress would mitigate visuomotor impairment. Secondly, we hypothesized dehydration would alter brain structures by expanding ventricular volume as observed in some (but not all) previous studies due to fluid movements across compartments, and these changes would be associated with reduced visuomotor performance.

## Methods

### Participants

All procedures and protocols were approved by the Georgia State University‐Georgia Institute of Technology Joint Advanced Brain Imaging Institutional Review Board and conformed to the guidelines set forth in the *Declaration of Helsinki*. Informed and written consent were obtained voluntarily by all participants before participation. Thirteen right‐handed (six female) healthy adults (age: 23.6 ± 4.2 year, body mass: 61.3 ± 6.0 kg, body fat: 15.2 ± 3.0%) participated in the study. Subjects were excluded if they were taking any medication, had any neurological condition, or metal in their body. Although fitness status was not assessed, all subjects were recreationally active, engaged in regular exercise (≥4 day/week). All subjects served as their own control and completed each condition.

### Experimental design

Thirteen subjects completed three preliminary sessions and three experimental trials (within ~2 weeks). Subjects were tested in Atlanta, GA, during the nonsummer months of either March–May or October–December (and therefore were assumed to not be heat acclimated). Female subjects were tested during the follicular phase of the menstrual cycle (or for *n* = 2, the first 10 days of their oral contraceptive pill pack) based on self‐reported history. Before all sessions, subjects were instructed to consume liberal (>500 mL) and consistent fluid the night before, abstain from alcohol and caffeine for the previous 12 h, and enter the laboratory after an overnight fast. During the 24 h prior to testing, subjects were asked to avoid heat exposure and exercise beyond normal daily activities. Three preliminary sessions were conducted to establish baseline body mass (BM), plasma osmolality (POsm), and urine specific gravity (USG; *ATAGO USA, Bellevue, WA*) as previously recommended (Cheuvront and Kenefick 2014). During one preliminary session, an exercise‐heat bout was completed by subjects to estimate sweat rate and verify workload (treadmill velocity and grade) for the experimental trials.

Following the preliminary sessions, subjects completed three experimental trials: control (CON; no exercise‐heat stress), exercise‐heat stress with fluid replacement (EHS), and exercise‐heat stress with dehydration (EHS‐DEH; exercise‐heat stress without fluid replacement). The order of experimental trials (EHS and EHS‐DEH) was counterbalanced, but CON usually occurred first (*n* = 7) due to scheduling constraints in the MRI facility. The experimental trials were initiated in the morning (~0700) and first morning BM, USG (<1.021), and POsm (<290 mOsm/kg) (Cheuvront et al. 2010) were assessed to ensure adequate hydration status (≤1% difference in BM from preceding 3 days average) (Cheuvront et al. 2010). Subjects then consumed a nutrition bar (250 kcal) and water (150 mL) 20 min before entering the hot (EHS, EHS‐DEH; 45°C, 15% RH) or temperate (CON; 22°C, 30% RH) environments. For EHS and EHS‐DEH, the exercise mirrored previous exercise‐heat stress protocols (Francesconi et al. 1983; Sawka et al. 1985; Cheuvront et al. 2010; Ely et al. 2013) consisting of 150 min (45 min walk/15 min rest cycles) on a treadmill at ~3.5 mph, 5% grade. This method using an intermittent exercise protocol with recovery periods (including 5 min in a cool room to obtain hourly body mass throughout) was selected to elicit dehydration (3% BM loss) at modest HR (initial HR of ~120 bt/min) while minimizing excessive elevations in core temperature as demonstrated previously (Ely et al. 2013). During EHS, subjects consumed a volume of water equivalent to sweat loss, while no water was consumed during EHS‐DEH (only mouth rinse permitted once per hour). Following EHS and EHS‐DEH, subjects had a 30 min recovery (moved to the temperate environment with cold packs applied to skin) prior to a final BM, blood glucose and POsm. Subjects then showered, changed into dry clothes, and were transported to the MRI facility (~5 min away) with a total recovery period of 45 min. This extended recovery/shower period was similar to one previously employed (Ely et al. 2013) to decrease skin and core temperatures. During CON, subjects reported under the same baseline conditions with meal provided but instead of walking in the heat, sat quietly for ~1.5 h in the temperate environment while abstaining from mentally stimulating activities before being transported to the MRI for scanning at the same time of day (~1100).

### Physiological and perceptual measures

During all exercise trials, heart rate (HR) and rectal temperature (*YSI, Yellow Springs, OH*) were measured at 5 min intervals, and did not exceed 90% of age‐predicted HR_max_ (220‐age) or 39.5°C, respectively. Blood samples were obtained by finger puncture on a heated digit after being seated for 10 min. POsm was determined from the median of at least three measurements (median of five if variation exceeded 1%) using freeze point depression (*Osmette II, Precision Systems, Natick, MA*) as described previously (Wittbrodt et al. 2015a). Blood glucose was measured (*OneTouch UltraMini, LifeScan Inc., Wayne, PA*) postexercise (~3 h after the meal) for EHS‐DEH and EHS and ~ 90 min after the meal for CON. Nude, dry BM was measured before and after each hour period of exercise on a digital platform scale and corrected for urine output. During EHS‐DEH, subjects were blinded to their BM. Rating of perceived exertion (RPE) (Borg 1982) and thirst (1–10 Likert scale) were also assessed at 5 min interval.

### MRI scanning and visuomotor task

Subjects were placed in the 3T MRI (*Siemens Trio, Siemens, Germany*) scanner with the 12‐channel head coil affixed and head position in a way to minimize movement in the *X*,* Y*, or *Z* axes. The scanning sequence consisted of a T1‐MPRAGE with 256 slices and 1.0 × 1.0 × 1.0 mm voxel size (TA: 6.17 sec, 9° flip angle, TI: 850 msec, TR: 2250 msec; TE: 3.98 msec) and a T2 Space with 1.0 × 1.0 × 1.0 mm voxel size (TA: 4.43 sec, TR: 3200 msec, TE: 428 msec). In between the T1 and T2 scan, subjects completed the motor pacing task (described below) during which Blood‐Oxygen‐Level‐Dependent (BOLD) responses were measured using an echo‐planar imaging sequence with a total of 714 volumes (37 slides; Tr = 2000 msec, TE = 30 msec, flip angle = 90°, field of view = 204 × 204 mm^2^, in‐plane resolution of 3 × 3 mm^2^, slice thickness: 3.0 mm). Each fMRI scanning (*n* = 2) block lasted approximately 11 min.

During the functional MRI (fMRI) scanning, subjects completed a visuomotor pacing task (VMPT; E*Prime, Psychology Software Tools, Sharpsburg, PA) requiring visually‐paced rhythmic finger tapping with the right index finger. In the scanner, subjects lay supine and viewed a display monitor (*Silent Vision 6011, Avotec, Stuart, FL*) via a mirror placed on the head coil. Headphones (*Silent Scan 3100, Avotec, Stuart, FL*) were placed on the subject and adequate visibility of the monitor was confirmed before each scan. If required, vision was corrected using MRI‐compatible lenses. Due to budget restrictions, several subjects (*n* = 3) did not undergo MRI scanning, and instead completed the VMPT in a MRI simulator (*Psychology Software Tools, Sharpsberg, PA*) built to mimic conditions within the MRI scanner (i.e., supine position, enclosed space, head coil). Thus, full behavioral data were obtained on 13 subjects, but fMRI imaging (for anatomical and BOLD responses) on only 10.

The VMPT consisted of 1 Hz alternating stimuli (yellow square presented for 500 msec) and fixation crosses (i.e., interstimulus interval) with two pacing variations: (1) regularly paced (VMPTr; fixation cross for 500 msec) and (2) irregularly paced (VMPTi; fixation cross presented for 400–600 msec). Subjects were instructed to respond to the stimulus (yellow square) by pressing a button box (*FORP 4 Diamond, Current Designs, Philadelphia, PA*). Errors were encoded binomially: “0” (missed response) or “1” (correct response). Blocks of thirty stimuli (all either VMPTr or VMPTi) were followed by 30 sec rest. Twenty total blocks were completed (*n* = 600 stimuli) with extended (120 sec) rest periods every five blocks, with total test duration equaling ~22 min. Block presentation (VMPTr or VMPTi) was randomized for each test iteration. Two behavioral measures were examined: accuracy (percentage correct responses) and reaction time (latency from stimulus presentation to button press). The nature of the VMPT dictated that only correct responses could be examined for reaction time. Both reaction time and accuracy were averaged across 5‐min blocks of time. Previous research has identified tasks with parameters of the VMPT (i.e., 1 Hz single digit finger tapping with one stimulus‐response combination) are not affected by learning or trial order (Bove et al. 2007).

### Anatomical analyses

Cortical reconstruction and volumetric segmentation were performed using the Freesurfer image analysis pipeline (*surfer.nmr.mgh.harvard.edu*). Briefly, the pipeline involves cortical surface extraction and “skull‐stripping” (removal of extracerebral voxels), gray/white matter segmentation based on intensity differences and geometric structures, computing planes to anatomically disconnect the two hemispheres and subcortical structures, computing a pial surface (smooth gray‐white matter interface), and correcting inter‐individual topological defects in surface by computing a spherical topology (Dale et al. 1999; Fischl et al. 1999). Furthermore, because dura and gray matter are difficult to distinguish with a T1 image, a T2 scan was provided to refine the estimate of the pial surface and registered with the T1 image using boundary‐based registration (Greve and Fischl 2009) provided as part of FreeSurfer (*surfer.nmr.mhg.harvard/fswiki/FsFast*). The Freesurfer anatomical pipeline is capable of detecting submillimeter differences between groups and has an inter‐class correlation of >0.95 and reproducibility >0.99 in measuring lateral ventricle volume (Kempton et al. 2011b). Because of large inter‐individual variability and sex‐specific differences in some brain regions (e.g., lateral ventricles) (Allen et al. 2002; Pletzer et al. 2010), a relative change (compared to CON) was computed for each area (% change = (([trial]−CON)/CON)*100).

### BOLD analysis

fMRI data analysis was completed using FSL (http://www.fmrib.ox.ac.uk/fsl) (Smith et al. 2004). All data were preprocessed by motion correcting images (MCFLIRT) (Jenkinson et al. 2002), removing non‐brain tissue (BET) (Smith 2002), distortion‐corrected with a fMRI field map using PRELUDE and FUGE (Smith et al. 2004), spatially smoothed using a Gaussian kernel of 8 mm full‐width half maximum, and high pass temporal filtering (sigma = 100 sec). First level fixed effects (time‐series) analysis was completed with a generalized linear model (FILM) including nonparametric estimation of time series autocorrelation (Woolrich et al. 2001). fMRI data for each subject were analyzed in native space (i.e., individual subject brain) before being initially registered to their own high resolution structural image and then subject to a nonlinear registration to standard MNI space (MNI152, Montreal Neurological Institute; Montreal, Quebec, Canada) using FNIRT. All BOLD signals were measured as signal intensity compared to the rest periods. Time series analysis was completed with the contrasts of the entire task (VMPTr and VMPTi).

Second‐level analyses (i.e., across subjects and sessions) were completed using mixed effects (FLAME 1+2) which uses Markov Chain Monte Carlo sampling to identify true random‐effect variance and degrees of freedom for each voxel. The main analysis examined the BOLD responses of the VMPT during each session and was restricted to gray matter voxels. To compare across sessions, *Z*‐statistic images were produced by applying a cluster threshold of *Z* ≥ 2.5 and (corrected) cluster significance threshold of *P* = 0.01 (Worsley et al. 1992). Contiguous clusters were identified using the *Z* statistic and then compared with the cluster probability threshold. Significant clusters were binarized and fed into the atlas query function of FSL. Cluster peaks and anatomical locations were localized in MNI152 space using the Lateralized Harvard‐Oxford Cortical Structural Atlas within the cortex and Harvard‐Oxford Subcortical Structural Atlas for subcortical structures. Statistical maps were overlaid onto a standard brain template using MRIcron (Rorden et al. 2007) with a threshold of *Z* ≥ 2.5. In addition, BOLD responses required for task completion were visualized during CON with a threshold of *Z* ≥ 1.6.

### Statistical analysis

All power analyses were computed to detect power of 0.8 at an alpha level of 0.05 using a repeated measures ANOVA with three comparisons (CON, EHS, EHS‐DEH). For brain anatomical changes, a power analysis was conducted using data from a previous study examining lateral ventricular volume changes following ~2% BM loss (Kempton et al. 2009). Using the reported effect size (0.37) between control and dehydration conditions along with a correlation among measures of 0.8 (given the high reliability as previously described), it was determined that a sample size of *n* = 7 was required (G*Power 3.1.9.2, gpower.hhu.de). For cognitive performance changes, 11 subjects were required based upon the calculated effect size (0.43) from previous motor coordination task data following ~3% BM loss (Cian et al. 2000) compared to an EHS trial (estimated within‐measures correlation of 0.5).

Physiological and perceptual variables (POsm, BM, USG, thirst, RPE, core temperature) were analyzed using a mixed model with repeated measures of trial (CON, EHS, EHS‐DEH) and time (e.g., pre, post) within the *nlme* package of R (*cran.r‐project.org/web/packages/nlme*). Relative change in brain areas were analyzed using a mixed model with repeated measure of trial. VMPT accuracy and reaction time were analyzed using a mixed model with repeated measures of trial, task version (VMPTr, VMPTi), and time block (first and last 5 min). For physiological, perceptual, visuomotor performance, and brain structural changes, if a significant main or interaction effect was observed, post‐hoc contrasts using Bonferroni‐Holm corrections were calculated using the *lsmeans* package in R (*cran.r‐project.org/web/packages/lsmeans*). Associations between changes in brain structures and plasma osmolality/VMPT were computed using one‐tail (negative association) Pearson product‐moment correlation coefficients. The alpha level was set a priori as *P* ≤ 0.05 to indicate statistical significance. Data are presented as mean ± 95% Confidence Interval, or 1.96 × SEM.

## Results

### Physiological changes

Baseline hydration status was similar across all trials with no differences in USG (CON: 1.017 ± 0.003, EHS: 1.020 ± 0.003, EHS‐DEH: 1.016 ± 0.003; *P* = 0.86) or POsm (CON: 283.8 ± 2.7, EHS: 283.6 ± 2.6, EHS‐DEH: 284.5 ± 2.2 mOsm/kg; *P* = 0.36). Baseline BM was also consistent across trials (*P* = 0.11). EHS‐DEH elicited significantly greater (*P* < 0.001) BM loss (−2.8 ± 0.3%) compared to EHS (−0.2 ± 0.3%) and was similar (*P* = 0.46) for men (−2.9 ± 0.4%) and women (−2.7 ± 0.4%). POsm following EHS‐DEH (293.8 ± 2.2 mOsm/kg) was greater (*P* < 0.001) compared to EHS (280.6 ± 2.6) and CON (283.8 ± 2.7 mOsm/kg) and EHS was lower compared to CON (*P* = 0.03). Furthermore, POsm increased (by 9.3 ± 2.1 mOsm/kg; *P* < 0.001) from baseline during EHS‐DEH and decreased during EHS (by 3.0 ± 2.1; *P* = 0.02). Final core temperature during exercise‐heat stress was not different (*P* = 0.10) between EHS‐DEH (38.6 ± 0.2) and EHS (38.3 ± 0.2°C), a rise of 1.6 ± 0.2 and 1.4 ± 0.2°C, respectively. Final RPE (EHS: 12.1 ± 0.9, EHS‐DEH: 12.9 ± 0.8) indicated exercise was considered “fairly light” to “somewhat hard” and not different between trials (*P* = 0.24). Postexercise rating of thirst sensation was higher in EHS‐DEH (5.8 ± 0.5) compared to EHS (1.4 ± 0.5, *P* < 0.0001). Postexercise blood glucose was >4.0 mmol/L in all subjects, with no significant differences between exercise trials (EHS: 5.3 ± 0.8, EHS‐DEH: 6.2 ± 0.7 mmol/L), although CON (7.0 ± 0.8 mmol/L) was higher (*P* = 0.001) than EHS but not different from EHS‐DEH.

### Visuomotor performance: reaction time

Testing order across all three trials did not affect VMPT reaction time (Trial 1: 165.4 ± 27.4, Trial 2: 160.8 ± 27.0; Trial 3: 152.3 ± 26.9 msec; *P* = 0.23). Reaction time was slower for irregularly paced (VMPTi) versus regularly paced (VMPTr) intervals (by 16.6 ± 7.2 msec, *P* < 0.001) and slower over time during the first 5 min block compared to the last 5 min block (by 22.3 ± 7.3 msec, *P* < 0.001). However, reaction time was not different among trials (CON: 168.9 ± 26.4, EHS: 159.9 ± 26.9, EHS‐DEH: 160.2 ± 24.8 msec; *P* = 0.54).

### Visuomotor performance: accuracy

**Figure 1:**
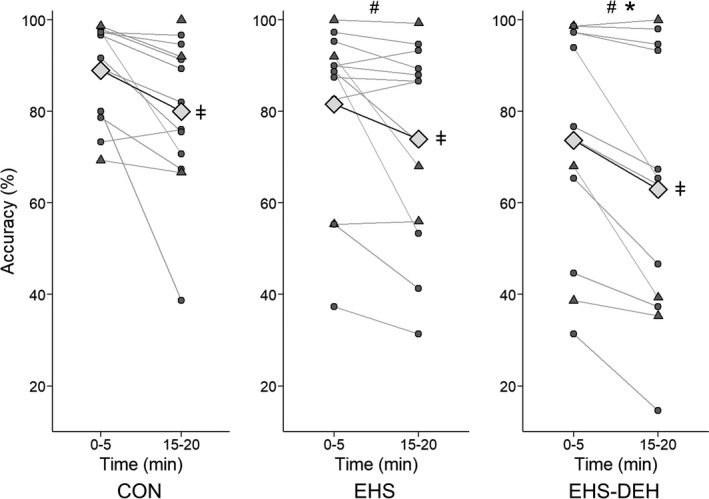
Mean accuracy (%) and individual responses (circles) for first and last 5 min time blocks of the visuomotor pacing task during resting control (CON), exercise heat stress with fluid replacement (EHS), and exercise heat stress coupled with dehydration (EHS‐DEH;* n* = 13). Triangle shapes indicate subjects scanned in the mock MRI scanner. Symbols above mean (diamond) indicate trial effect (^#^ *P* < 0.05 vs. CON, *P < 0.05 vs. EHS) and symbols in the right margin indicate a time effect (^ǂ^ *P* < 0.05 15–20 min lower than 0–5 min).

Testing order across all three trials did not affect VMPT accuracy (Trial 1: 83.5 ± 9.2, Trial 2: 79.7 ± 10.9; Trial 3: 78.6 ± 10.5%; *P* = 0.65). Accuracy was not different based on the test pacing intervals (VMPTr: 78.5 ± 13.8%, VMPTi: 75.7 ± 11.1%, *P* = 0.09) or a pacing interval by trial interaction (*P* = 0.28). Therefore, VMPT data were pooled across regular and irregular paced tasks. Over 20 min, accuracy was lower during EHS‐DEH (69.7 ± 13.5%) compared to CON (85.1 ± 7.0%, *P* = 0.008) and EHS (77.9 ± 10.3%; *P* = 0.02). Accuracy was also lower during EHS compared to CON (*P* = 0.03). Figure 1 presents VMPT accuracy during the first and last 5‐min time blocks across trials. As early as 5 min, EHS‐DEH impaired accuracy compared to both CON (by −15.4 ± 9.3%, *P* = 0.003) and EHS (by −6.9 ± 6.2%, *P* = 0.03), and EHS compared to CON (by −8.5 ± 6.4%, *P* = 0.02). Accuracy was reduced over time from the first 5 min block compared to the last 5 min (by 9.2 ± 3.3%, *P* < 0.0001), although no time by trial interaction was observed (*P* = 0.58).

### Brain anatomical changes

Total brain volume (all tissues including cerebellum and ventricles but excluding the dura mater) was obtained from a data set of *n* = 10 (five females) and not different among trials (*P* = 0.26). Total intracranial volume (total brain volume and sinus areas) was not different between CON and EHS‐DEH (*P* = 0.96); but increased during EHS versus CON (by 1.49 ± 0.8%, *P* < 0.0001). The following brain structures were not significantly (*P* > 0.05) altered by the experimental trials: total gray matter, cortical gray matter, cerebellar white matter, nucleus accumbens, amygdala, caudate, hippocampus, putamen, ventral dorsal column, brain stem, and choroid plexus.

Table 1 provides the brain structure changes associated with the experimental trials. EHS (vs. CON) increased (*P* < 0.05) volumes of the cortical white matter, cerebellum, globus pallidus, and cerebellar gray matter (between ~1 and ~5%); but decreased ventricular volume and nonventricular cerebral spinal fluid (between 5 and 6%). In direct contrast to this, EHS‐DEH (vs. CON) increased volume of the ventricles and nonventricular cerebral spinal fluid (by 6.0–6.8%), but also decreased volume of the cerebellum (by 0.7%), subcortical gray matter (by 1.1%), and thalamus (by 2.7%). In addition, EHS‐DEH (vs. EHS) decreased volume of cortical white matter, subcortical gray matter, cerebellum, cerebellar gray matter, and corpus callosum.

**Table 1:** Mean ± 95% CI brain anatomical changes associated with the experimental trials (*n* = 10)

	EHS versus CON (% Change)	EHS‐DEH versus CON (% Change)	EHS‐DEH versus EHS (% Change)
Aggregate brain areas			
Cortical white matter	1.2 ± 1.1*	0.1 ± 0.9	−1.1 ± 0.9*
Subcortical gray matter	0.5 ± 0.9	−1.1 ± 0.9*	−1.5 ± 0.9*
Brain structures			
Cerebellum	1.5 ± 0.8*	−0.7 ± 0.8*	−2.2 ± 0.8*
Cerebellar gray matter	1.9 ± 0.9*	−0.7 ± 0.9	−2.5 ± 1.0*
Corpus callosum	0.5 ± 1.0	−0.8 ± 0.9	−1.3 ± 0.9*
Subcortical gray matter			
Globus pallidus	5.2 ± 5.1*	1.4 ± 3.3	−3.8 ± 3.9
Thalamus	1.1 ± 1.7	−2.7 ± 1.3*	−3.8 ± 1.7*
Ventricular system			
All Ventricles	−5.3 ± 1.7*	6.8 ± 3.4*	12.1 ± 2.5*
Lateral Ventricles	−5.0 ± 2.0*	7.5 ± 3.5*	12.5 ± 2.7*
Third and Fourth Ventricles	−8.9 ± 4.3*	3.8 ± 3.9	12.8 ± 4.0*
Nonventricular Cerebrospinal Fluid	−6.2 ± 2.9*	6.0 ± 5.4*	12.2 ± 4.4*

Areas are expressed as a relative change from resting control (CON) for exercise heat stress without dehydration (EHS) and exercise heat stress with dehydration (EHS‐DEH). *P ≤ 0.05; ND, no significant difference (*P* > 0.05).

**Figure 2:**
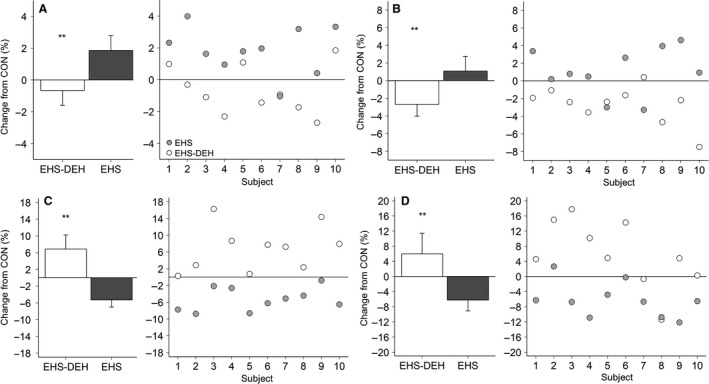
Left panel (A–D) Mean (±95% Confidence Interval) relative change (from resting control; CON) within cerebellar gray matter (A), thalamus (B), all ventricles (C), and nonventricular cerebrospinal fluid (D), during exercise heat stress (EHS) and exercise heat stress coupled with dehydration (EHS‐DEH). Right panel within (A–D) Relative changes for each individual subject (*n* = 10) following EHS (filled circles) and EHS‐DEH (open circles). *P < 0.05, **P < 0.01 versus EHS.

Figure 2 provides the mean and individual data for selected anatomical sites (cerebellar gray matter, thalamus, all ventricles, nonventricular CSF) to illustrate this dichotomy between trials. EHS consistently increased cerebral gray matter and thalamus volume (assumed fluid gain). EHS‐DEH consistently decreased tissue volume (assumed fluid loss) concomitant with ventricular volume changes in the opposite direction (i.e., reduction with EHS, presumably as surrounding tissues gained fluid, and expansion with EHS‐DEH, as surrounding tissues crenate from fluid loss).

**Figure 3:**
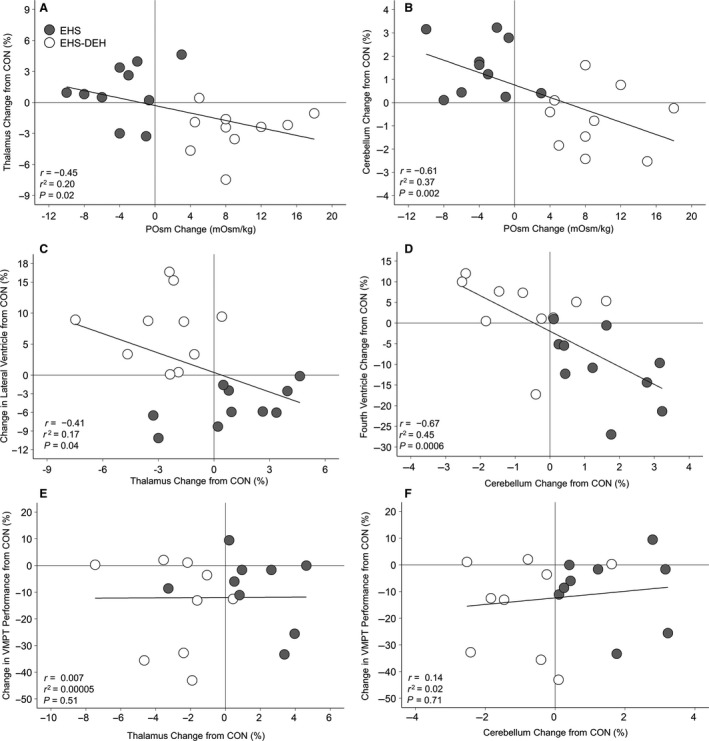
Association between changes in plasma osmolality (from preexercise baseline) and brain structure changes (relative to resting control; CON) in thalamus (A) and cerebellum (B). Associations between relative changes (from CON) in the thalamus (C) and cerebellum (D) with interfacing ventricular structure. Associations between relative changes (from CON) in the thalamus (E) and cerebellum (F) with mean change (from CON) in visuomotor task (VMPT) accuracy. Circles indicate individual data (*n* = 10) by trial: exercise heat stress with fluid replacement (EHS, closed circles) and exercise‐heat stress without fluid replacement (EHS‐DEH, open circles).

Figure 3A–D presents the relationship between changes in specific brain structures (thalamus, cerebellum) with their adjacent ventricles and change in POsm. Change in POsm was inversely correlated with changes in cerebellum (*r* = −0.61, *P* = 0.005), cerebellar gray matter (*r* = −0.63, *P* = 0.003), and thalamus (*r* = −0.45, *P* = 0.04) volumes but directly associated with changes in total ventricular (*r* = 0.74, *P* = 0.0002) and nonventricular cerebrospinal fluid volumes (*r* = 0.70, *P* = 0.0006). Moreover, lateral ventricle (*r* = −0.41; *P* = 0.04) and fourth ventricle (*r* = −0.67; *P* = 0.0006) expansion were significantly associated with reductions in thalamus and cerebellum volume, respectively. Figure 3E–F present a scatter plot illustrating the nonsignificant (*P* > 0.50) relationships between changes in thalamus and cerebellum volume and changes in VMPT accuracy. VMPT accuracy was also not associated with changes in POsm (*r* = 0.08, *P* = 0.38).

### Brain function via BOLD during VMPT

**Figure 4:**
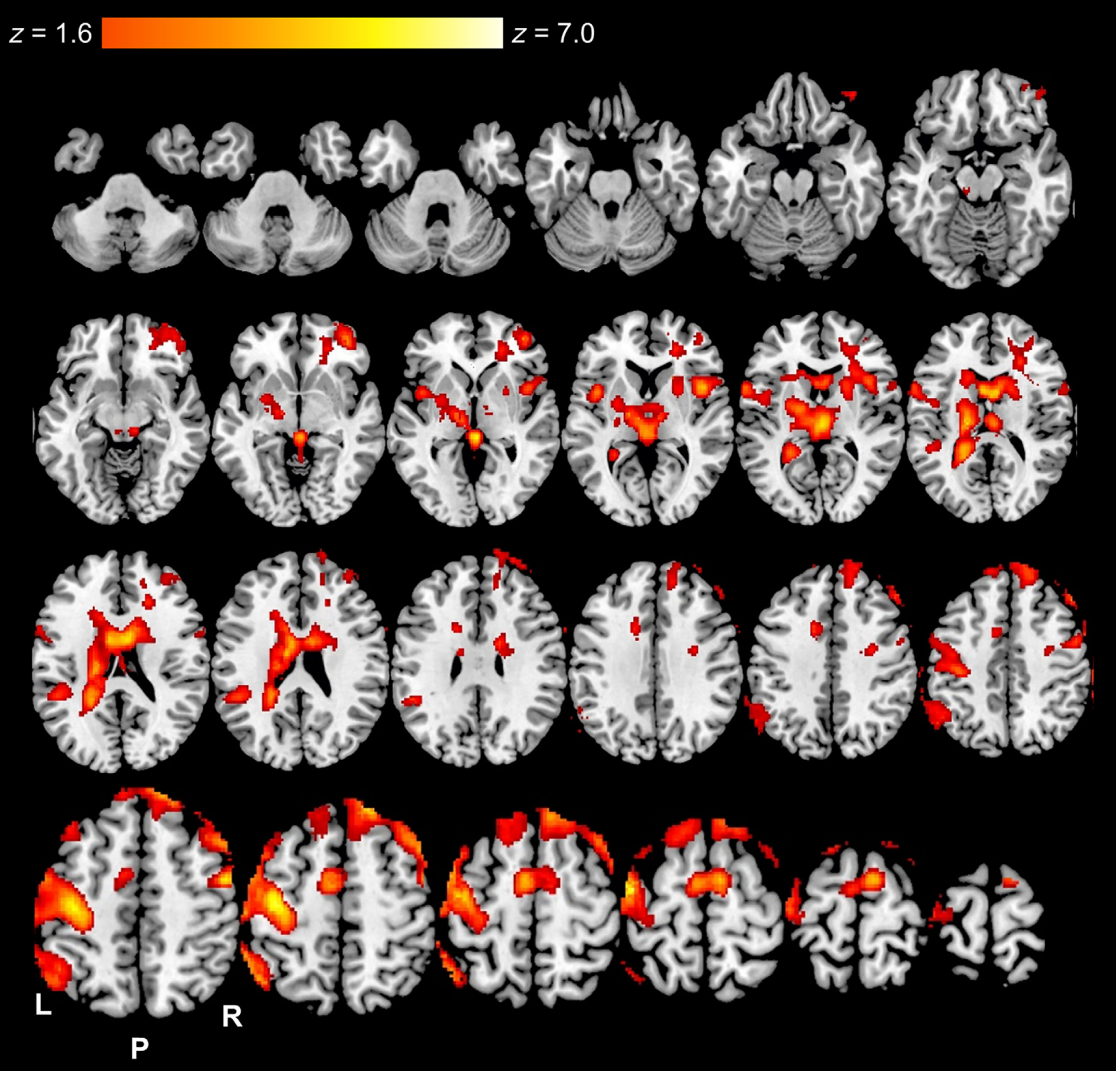
Axial slices of significantly (*Z* ≥ 1.6 with cluster correction of *P* < 0.05) elevated Blood‐Oxygen‐Level‐Dependent (BOLD) responses throughout the entire visuomotor pacing task during the resting control (CON) trial (*n* = 10). Color gradient indicates level of elevated BOLD responses.

Figure 4 presents BOLD responses obtained from a data set of *n* = 10 (five females) during CON to illustrate the task‐dependent neural resource requirements during the entire 20‐min VMPT (combining regular and irregular variations). Elevated BOLD responses were observed for the following brain areas: left sensorimotor, bilateral supplementary motor, bilateral thalamus, bilateral putamen, bilateral caudate, right frontal pole, bilateral middle and superior frontal gyri, supramarginal gyrus, angular gyrus, left frontal, central, and right operculum, and bilateral insula.

**Figure 5:**
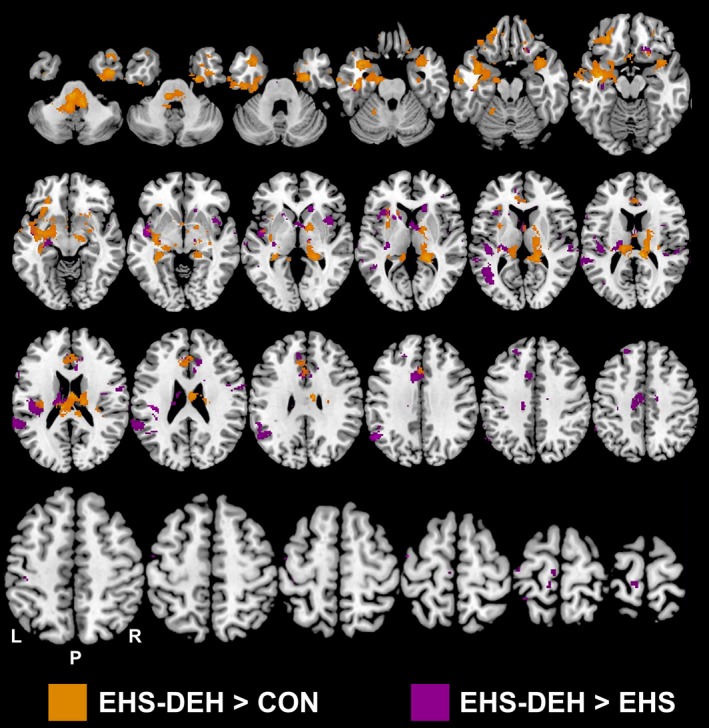
Significantly elevated (*Z* ≥ 2.5 with cluster correction of *P* < 0.01) Blood‐Oxygen‐Level‐Dependent (BOLD) responses for the entire visuomotor pacing task during exercise heat stress with dehydration (EHS‐DEH) compared to resting control (CON) and exercise heat stress without dehydration (EHS;* n* = 10). Areas of colour indicate locations where EHS‐DEH elicited greater BOLD responses compared to CON or EHS. No differences were observed between CON and EHS (*P* > 0.05).

BOLD response contrasts for the entire 20‐min are presented in Table 2 and Figure 5. No differences in BOLD responses were observed between EHS and CON. However, EHS‐DEH elicited two elevated BOLD clusters (*P* < 0.05) compared to CON. The first cluster was located within the bilateral thalamus, bilateral caudate, right putamen, right pallidum, right parietal lobe, right insula, and anterior cingulate cortex while the second cluster was located within the left temporal lobe, left frontal lobe, left putamen, left amygdala, left hippocampus, and left insular cortex. EHS‐DEH also elevated BOLD responses (*P* < 0.05) versus EHS within the bilateral frontal lobe, bilateral thalamus, bilateral caudate, left central operculum, left insular cortex, anterior cingulate cortex, and supplementary motor area (Table 2, Figure 5).

**Table 2:** Significantly elevated (*Z* ≥ 2.5) BOLD responses observed throughout the entire visuomotor pacing task for exercise heat stress with dehydration (EHS‐DEH) compared to resting control (CON) and exercise heat stress without dehydration (EHS; *n* = 10)

	Regions in cluster	Hemisphere
EHS versus CON	No significant clusters	
EHS‐DEH > CON		
Cluster 1 Peak: 22, −40, 6	*Caudate*	*Right*
Voxels: 2105	*Caudate*	*Left*
Peak Z: 4.4	Cingulate Gyrus, Anterior	Right
	Cingulate Gyrus, Posterior	Right
	Hippocampus	Right
	*Insular Cortex*	*Right*
	Pallidum	Right
	Parietal Lobe, Operculum Cortex	Right
	*Putamen*	*Right*
	Subcallosal Cortex	
	*Thalamus*	*Right*
	*Thalamus*	*Left*
Cluster 2 Peak: −38, −8, −12	Amygdala	Left
Voxels: 2505	Frontal Lobe, Inferior Gyrus	Left
Peak Z: 4.2	*Frontal Lobe, Operculum Corte*	*Left*
	Frontal Lobe, Orbital Cortex	Left
	Frontal Lobe, Pole	Left
	Hippocampus	Left
	*Insular Cortex*	*Left*
	*Putamen*	*Left*
	Temporal Lobe, Fusiform Cortex	Left
	Temporal Lobe, Inferior Gyrus	Left
	Temporal Lobe, Pole	Left
	Temporal Lobe, Planum Polare	Left
EHS‐DEH > EHS		
Cluster Peak: −20, 16, 4	*Caudate*	*Right*
Voxels: 1905	*Caudate*	*Left*
Peak Z: 4.1	*Central Operculum Cortex*	*Left*
	Cingulate Gyrus, Anterior	Left
	Cingulate Gyrus, Posterior	*Left*
	*Frontal Lobe, Operculum Cortex*	*Right*
	Frontal Lobe, Pole	*Left*
	*Frontal Lobe, Middle Frontal Gyrus*	*Left*
	*Frontal Lobe, Middle Frontal Gyrus*	Left
	*Frontal Lobe, Superior Frontal* Gyrus	*Left*
	*Insular Cortex*	*Left*
	Paracingulate Gyrus	*Right*
	*Putamen*	*Left*
	*Supplementary Motor Area*	
	*Thalamus*	*Left*
	*Thalamus*	*Right*

Cluster peaks are presented in MNI152 coordinates. Italicized areas indicate task‐specific areas as identified during CON.

## Discussion

Dehydration is a common perturbation in occupational, military, and athletic populations who perform cognitive‐motor tasks. Early research observed dehydration alters multiple cognitive‐motor domains (Sharma et al. 1986; Gopinathan et al. 1988), however, many subsequent studies have not supported this position (Adam et al. 2008; Ely et al. 2013; van den Heuvel et al. 2017) and potential mechanisms responsible remain elusive. Our data uniquely addressed if changes in brain structures due to dehydration (at levels known to induce physiological compensation) were aligned with performance impairments and altered neural processing during a fundamental, prolonged visuomotor task. Visuomotor timing, a core tenant of visuomotor function (Buhusi and Meck 2005), was selected, as it is a critical component for occupational, military, and athletic tasks at a required motor output at ~1 Hz, the frequency at which human motor control operates (Buhusi and Meck 2005).

Our major finding was that exercise‐heat stress (despite water ingestion matching sweat loss) significantly impaired visuomotor performance by 8% which was further exacerbated with dehydration by another 8%. Therefore, we accept our hypothesis that dehydration impairs visuomotor performance. Our recent meta‐analysis identified that visuomotor performance is impaired by dehydration (Wittbrodt and Millard‐Stafford 2018), however not all individual studies have supported this finding (Cian et al. 2001) despite similar body water deficits. Interestingly, visuomotor impairments were demonstrated within the initial 5 min of our timed finger‐tapping task. The VMPT, although a “simple” task not requiring executive or higher‐order processing, is a prolonged monotonous task containing only one stimulus, thus characteristic of tasks which stress attentional capacity (Sturm and Willmes 2001). Although dehydration per se did not alter the rate of performance decline over time (~9% over the 20 min across all trials including resting control), we cannot exclude the possibility that performance impairments following both exercise‐heat stress trials are partially explained by a diminished capacity of visual‐spatial resources.

Our study corroborates others observing that dehydration does not reduce total brain volume (Kempton et al. 2009, 2011a; Watson et al. 2010; Meyers et al. 2016), but provides novel information regarding changes to brain structures. Similar to our data, two previous studies observed lateral ventricles expansion following dehydration (Kempton et al. 2009, 2011a), however, others reported either no change (Duning et al. 2005; Streitburger et al. 2012; Meyers et al. 2016) or decreases in volume (Watson et al. 2010). Previously, disparate mechanisms have been presented to explain ventricular volume change following dehydration either: (1) ventricular shrinkage as a consequence of overall hypovolemia (Watson et al. 2010) or (2) ventricular expansion via an *ex vacuo* mechanism resulting from osmotic gradients drawing fluid out of the intracellular spaces (Kempton et al. 2009). Our findings support the *ex vacuo* expansion of the ventricular system during dehydration with hypotonic sweat. POsm increases also may elevate cerebrospinal fluid osmolality (Pape and Katzman 1970; Szczepańska‐Sadowska et al. 1984), as the choroid plexus secretes a fluid isotonic to blood plasma (DiMattio et al. 1975). Hypertonic cerebrospinal fluid decreases choroid plexus bulk flow, potentially to maintain total brain volume, as evidenced by shrinking total brain volume, although only reported with severely elevated cerebrospinal fluid osmolality (+45 mOsm/kg) (DiMattio et al. 1975). This mechanism likely explains why total brain volume was unchanged following hypertonic hypovolemia (POsm elevation) but ventricular volume/nonventricular cerebrospinal fluid expanded and were associated with shrinkage of the periventricular structures.

In contrast to our hypothesis, when preventing dehydration during exercise in the heat (water replacement to match 100% sweat loss), plasma osmolality was reduced (~3 mOsm/kg) with the unexpected finding of opposing changes to brain structures (e.g., ventricular volume constriction, periventricular expansion) compared to exercise‐heat stress without water replacement. Given the potential dilution effect of replacing sweat loss with plain water similar to other studies (Anastasiou et al. 2009), the cerebrospinal fluid osmolality may decrease in parallel. Hypotonic fluid perfusing the ventricular system will increase choroid plexus bulk flow, decreasing cerebrospinal fluid osmolality and expanding cortical gray and white matter (DiMattio et al. 1975), consistent with our finding. These results suggest replacing 100% of sweat loss with electrolyte‐free water may begin to induce brain swelling, which is consistent directionally with the effects of hyponatremia (Montain et al. 2001).

In contrast with our hypotheses, although both exercise‐heat stress with and without water replacement altered brain structure volumes, no clear association with visuomotor performance was observed. One might hypothesize that net tissue volume changes (in either direction), mediated by osmolality perturbations (as shown in Figure 3), could explain performance impairment since both cerebral edema (e.g., hyponatremia) and dehydration symptoms include deteriorated mental status (Adolph 1947; Hew‐Butler et al. 2017). However, this hypothesis is not supported by Figure 3E–F, as some individuals with the largest thalamus expansions and contractions demonstrated sustained visuomotor accuracy. Thus, mechanisms unrelated to brain structural changes are likely responsible for visuomotor impairments following dehydration. An alternative hypothesis underlying the impaired visuomotor performance might be explained by an acute stress response (i.e., increased catecholamine turnover) elicited by both exercise‐heat stress and body water deficits (Melin et al. 1988; Cheuvront et al. 2004). Heat stress is known to impact cognitive‐motor performance, but impairments previously appeared limited to testing while in the hot environment (Hancock et al. 2007) or with elevated core temperatures (~39.5°C) (Gaoua 2010; Piil et al. 2017). Dehydration also induces an acute stress response (Popova et al. 2001), and therefore visuomotor performance may, in part, have been impaired primarily via a graded (and possibly additive) stress response elicited by exercise‐heat stress alone and exacerbated by body water deficits. However, not all studies using exercise‐heat stress (some at 50°C) have found cognitive‐motor impairments following dehydration (Ely et al. 2013), in fact, with some reporting improved fine motor performance (Bandelow et al. 2010). Future studies on brain structure and function should consider including perceptual measures of task difficulty to discriminate between physical and mental demands and alternative dehydration methods (e.g. fluid restriction, diuretics) to understand the influence of heat stress per se.

Furthermore, we acknowledge large interindividual variability in visuomotor performance (four subjects had little change in accuracy across trials). Why some individuals were more prone to the effects of prior exercise‐heat stress both with and without dehydration, while others are resistant, is unclear. Vigilance for sustaining a simple task may be attributed to attentional differences, although no subjects were taking medication or diagnosed with attentional deficit deficiencies. When searching for other potential variables linked to the heterogeneity in responses, no single factor (e.g., sex, month tested, final core temperature, or RPE) appeared to explain the results. Unfortunately, individual data is not consistently presented in previous studies; thus, it is unclear whether our pattern of results is typical. Future work should attempt to investigate the source of individual variability as it may explain the overall lack of consistency in the literature.

Our exploratory brain activity analysis suggests dehydration elevates neural activity during a simple visuomotor task compared to resting control and exercise‐heat stress with water replacement. The increase in BOLD following dehydration is similar to a prior study with modest dehydration of 2% BM loss (Kempton et al. 2011a) reporting elevated neural activity in fronto‐parietal areas although executive function was maintained. This is in contrast with our results indicating elevated neural resources devoted to the task were unable to sustain visuomotor performance. Both studies assessed BOLD responses on relatively modest sample sizes (*n* = 10) and may be at risk for false positives and/or may be underpowered to return differences (e.g., for EHS vs. CON in this study). Elevated neural activity following dehydration in this study occurred in task‐specific areas (e.g., left thalamus, left putamen, and left caudate) but also in more ipsilateral areas (i.e., right thalamus/basal ganglia) suggesting body water deficits elicit greater neural demands during time keeping, planning and/or execution of finger tapping (Cerasa et al. 2004; Horenstein et al. 2009). Secondly, the elevated neural activity within the hippocampus, inferior, middle, and superior temporal lobes may represent altered visual processing following dehydration. The inferior temporal lobe integrates neural input from the primary visual cortex to encode necessary object information for motor‐specific visual processing (i.e., ventral visual stream) (Goodale and Milner 1992); the medial temporal lobe may integrate information between the ventral and dorsal processing streams (Tankus and Fried 2012). Alternatively, the elevated neural activity following dehydration may suggest additive neural demands of VMPT task completion and homeostasis maintenance, as evidenced by elevated brain activations within the anterior cingulate and superior temporal gyrus, brain areas known to be involved with signaling thirst (Egan et al. 2003). Furthermore, a right‐lateralized network involving both the anterior cingulate and thalamus mediates attentional resources (Sturm and Willmes 2001), which, when combined with the progressive decline in accuracy, could also partially explain increased ipsilateral neural activity following dehydration. As a result, this exploratory analysis suggests elevated brain activation following dehydration elicited by exercise‐heat stress may result from a confluence of sources.

In summary, our study is the first to analyze brain structures and assess fundamental visuomotor functioning (performance, brain function) at body water deficits that require homeostatic responses (Cheuvront et al. 2010, 2013). This study made the following novel observations: (1) visuomotor performance is impaired following exercise‐heat stress with water replacement and further exacerbated by dehydration; (2) several brain structure volumes are sensitive to both increases and modest decreases in plasma osmolality but these changes were unrelated to visuomotor performance; (3) dehydration may increase neural activity in task‐specific and nontask specific areas. These observations suggest dehydration may elicit fundamental impairments in the visuomotor system which could potentially impact military and occupation‐specific tasks requiring a prolonged motor output.

## Conflict of Interest

There are no conflicts of interest for any of the authors with regard to this study.

## References

[phy213805-bib-0001] Adam, G. , R. Carter , S. Cheuvront , D. Merullo , J. Castellani , H. Lieberman , et al. 2008 Hydration effects on cognitive performance during military tasks in temperate and cold environments. Physiol. Behav. 93:748–756. 18166204 10.1016/j.physbeh.2007.11.028

[phy213805-bib-0002] Adolph, E. 1947 Physiology of man in the desert, Pp. 137–141. Interscience New York, NY.

[phy213805-bib-0003] Allen, J. , H. Damasio , and T. Grabowski . 2002 Normal neuroanatomical variation in the human brain: an MRI‐volumetric study. Am. J. Phys. Anthropol. 118:341–358. 12124914 10.1002/ajpa.10092

[phy213805-bib-0004] Anastasiou, C. A. , S. A. Kavouras , G. Arnaoutis , A. Gioxari , M. Kollia , E. Botoula , et al. 2009 Sodium replacement and plasma sodium drop during exercise in the heat when fluid intake matches fluid loss. J. Athl. Train. 44:117–123. 19295955 10.4085/1062-6050-44.2.117 PMC2657026

[phy213805-bib-0005] Armstrong, L. E. , M. S. Ganio , D. J. Casa , E. C. Lee , B. P. McDermott , J. F. Klau , et al. 2012 Mild dehydration affects mood in healthy young women. J. Nutr. 142:382–388. 22190027 10.3945/jn.111.142000

[phy213805-bib-0006] Baker, L. B. , D. E. Conroy , and W. L. Kenney . 2007a Dehydration impairs vigilance‐related attention in male basketball players. Med. Sci. Sports Exerc. 39:976–983. 17545888 10.1097/mss.0b013e3180471ff2

[phy213805-bib-0007] Baker, L. B. , K. A. Dougherty , M. Chow , and W. L. Kenney . 2007b Progressive dehydration causes a progressive decline in basketball skill performance. Med. Sci. Sports Exerc. 39:1114–1123. 17596779 10.1249/mss.0b013e3180574b02

[phy213805-bib-0008] Bandelow, S. , R. Maughan , S. Shirreffs , K. Ozgünen , S. Kurdak , G. Ersöz , et al. 2010 The effects of exercise, heat, cooling and rehydration strategies on cognitive function in football players. Scand. J. Med. Sci. Sports 20:148–160. 21029202 10.1111/j.1600-0838.2010.01220.x

[phy213805-bib-0009] Borg, G. A. 1982 Psychophysical bases of perceived exertion. Med. Sci. Sports Exerc. 14:377–381. 7154893

[phy213805-bib-0010] Bove, M. , A. Tacchino , A. Novellino , C. Trompetto , G. Abbruzzese , and M. F. Ghilardi . 2007 The effects of rate and sequence complexity on repetitive finger movements. Brain Res. 1153:84–91. 17459347 10.1016/j.brainres.2007.03.063

[phy213805-bib-0011] Breteler, M. M. , N. M. van Amerongen , J. C. van Swieten , J. J. Claus , D. E. Grobbee , J. van Gijn , et al. 1994 Cognitive correlates of ventricular enlargement and cerebral white matter lesions on magnetic resonance imaging. The Rotterdam Study. Stroke 25:1109–1115. 8202966 10.1161/01.str.25.6.1109

[phy213805-bib-0012] Buhusi, C. V. , and W. H. Meck . 2005 What makes us tick? Functional and neural mechanisms of interval timing. Nat. Rev. Neurosci. 6:755–765. 16163383 10.1038/nrn1764

[phy213805-bib-0013] Cerasa, A. , G. E. Hagberg , M. Bianciardi , and U. Sabatini . 2004 Visually cued motor synchronization: modulation of fMRI activation patterns by baseline condition. Neurosci. Lett. 373:32–37. 10.1016/j.neulet.2004.09.076 15555772

[phy213805-bib-0014] Cheuvront, S. , and R. Kenefick . 2014 Dehydration: physiology, assessment, and performance effects. Compr. Physiol. 4:257–285. 24692140 10.1002/cphy.c130017

[phy213805-bib-0015] Cheuvront, S. N. , R. Carter , M. A. Kolka , H. R. Lieberman , M. D. Kellogg , and M. N. Sawka . 2004 Branched‐chain amino acid supplementation and human performance when hypohydrated in the heat. J. Appl. Physiol. 1985:1275–1282. 10.1152/japplphysiol.00357.2004 15358751

[phy213805-bib-0016] Cheuvront, S. N. , B. R. Ely , R. W. Kenefick , and M. N. Sawka . 2010 Biological variation and diagnostic accuracy of dehydration assessment markers. Am. J. Clin. Nutr. 92:565–573. 20631205 10.3945/ajcn.2010.29490

[phy213805-bib-0017] Cheuvront, S. N. , R. W. Kenefick , N. Charkoudian , and M. N. Sawka . 2013 Physiologic basis for understanding quantitative dehydration assessment. Am. J. Clin. Nutr. 30:455–462. 10.3945/ajcn.112.044172. 23343973

[phy213805-bib-0018] Cian, C. , N. Koulmann , P. A. Barraud , C. Raphel , C. Jimenez , and B. Melin . 2000 Influence of variations in body hydration on cognitive function: effect of hyperhydration, heat stress, and exercise‐induced dehydration. J. Psychophysiol. 14:29–36.

[phy213805-bib-0019] Cian, C. , P. A. Barraud , B. Melin , and C. Raphel . 2001 Effects of fluid ingestion on cognitive function after heat stress or exercise‐induced dehydration. Int. J. Psychophysiol. 42:243–251. 11812391 10.1016/s0167-8760(01)00142-8

[phy213805-bib-0020] Cisek, P. , and J. Kalaska . 2010 Neural mechanisms for interacting with a world full of action choices. Annu. Rev. Neurosci. 33:269–298. 20345247 10.1146/annurev.neuro.051508.135409

[phy213805-bib-0021] Coffey, C. E. , W. E. Wilkinson , L. Parashos , S. A. R. Soady , R. J. Sullivan , L. J. Patterson , et al. 1992 Quantitative cerebral anatomy of the aging human brain A cross‐sectional study using magnetic resonance imaging. Neurology 42:527. 1549213 10.1212/wnl.42.3.527

[phy213805-bib-0022] Dale, A. , B. Fischl , and M. Sereno . 1999 Cortical surface‐based analysis: I. Segmentation and surface reconstruction. NeuroImage 9:179–194. 9931268 10.1006/nimg.1998.0395

[phy213805-bib-0023] De Petris, L. , A. Luchetti , and F. Emma . 2001 Cell volume regulation and transport mechanisms across the blood‐brain barrier: implications for the management of hypernatraemic states. Eur. J. Pediatr. 160:71–77. 11271393 10.1007/s004310000631

[phy213805-bib-0024] Dickson, J. M. , H. M. Weavers , N. Mitchell , E. M. Winter , I. D. Wilkinson , E. J. R. Van Beek , et al. 2005 The effects of dehydration on brain volume ‐ preliminary results. Int. J. Sports Med. 26:481–485. 16037892 10.1055/s-2004-821318

[phy213805-bib-0025] DiMattio, J. , G. M. Hochwald , C. Malhan , and A. Wald . 1975 Effects of changes in serum osmolarity on bulk flow of fluid into cerebral ventricles and on brain water content. Pflüg Arch. 359:253–264. 10.1007/BF00587383 1103083

[phy213805-bib-0026] Duning, T. , S. Kloska , O. Steinstrater , H. Kugel , W. Heindel , and S. Knecht . 2005 Dehydration confounds the assessment of brain atrophy. Neurology 64:548–550. 15699394 10.1212/01.WNL.0000150542.16969.CC

[phy213805-bib-0027] Egan, G. , T. Silk , F. Zamarripa , J. Williams , P. Federico , R. Cunnington , et al. 2003 Neural correlates of the emergence of consciousness of thirst. Proc. Natl Acad. Sci. USA 100:15241–15246. 14657368 10.1073/pnas.2136650100 PMC299974

[phy213805-bib-0028] Ely, B. , K. Sollanek , S. Cheuvront , H. Lieberman , and R. Kenefick . 2013 Hypohydration and acute thermal stress affect mood state but not cognition or dynamic postural balance. Eur. J. Appl. Physiol. 113:1027–1034. 23064870 10.1007/s00421-012-2506-6

[phy213805-bib-0029] Falck, C. , and T. Scheffer . 1854 Untersuchungen uber den Wassergehalt der Organe durstender und nicht durstender Hunde. Arch. Physiol. Heilkd. 13:508–522.

[phy213805-bib-0030] Fischl, B. , M. I. Sereno , and A. M. Dale . 1999 Cortical surface‐based analysis: II: inflation, flattening, and a surface‐based coordinate system. NeuroImage 9:195–207. 9931269 10.1006/nimg.1998.0396

[phy213805-bib-0031] Fitts, P. M. 1954 The information capacity of the human motor system in controlling the amplitude of movement. J. Exp. Psychol. 47:381–391. 13174710

[phy213805-bib-0032] Francesconi, R. P. , M. N. Sawka , and K. B. Pandolf . 1983 Hypohydration and heat acclimation: plasma renin and aldosterone during exercise. J. Appl. Physiol. 55:1790–1794. 6363364 10.1152/jappl.1983.55.6.1790

[phy213805-bib-0033] Gaoua, N. 2010 Cognitive function in hot environments: a question of methodology. Scand. J. Med. Sci. Sports 20:60–70. 21029192 10.1111/j.1600-0838.2010.01210.x

[phy213805-bib-0034] Goodale, M. A. , and A. D. Milner . 1992 Separate visual pathways for perception and action. Trends Neurosci. 15:20–25. 1374953 10.1016/0166-2236(92)90344-8

[phy213805-bib-0035] Gopinathan, P. M. , G. Pichan , and V. M. Sharma . 1988 Role of dehydration in heat stress‐induced variations in mental performance. Arch. Environ. Health 43:15–17. 3355239 10.1080/00039896.1988.9934367

[phy213805-bib-0036] Greve, D. N. , and B. Fischl . 2009 Accurate and robust brain image alignment using boundary‐based registration. NeuroImage 48:63–72. 19573611 10.1016/j.neuroimage.2009.06.060 PMC2733527

[phy213805-bib-0037] Gullans, S. R. , and J. G. Verbalis . 1993 Control of brain volume during hyperosmolar and hypoosmolar conditions. Annu. Rev. Med. 44:289–301. 8476251 10.1146/annurev.me.44.020193.001445

[phy213805-bib-0038] Hancock, P. A. , J. M. Ross , and J. L. Szalma . 2007 A meta‐analysis of performance response under thermal stressors. Hum. Factors 49:851–877. 17915603 10.1518/001872007X230226

[phy213805-bib-0039] van den Heuvel, A. M. , B. J. Haberley , D. J. Hoyle , N. A. Taylor , and R. J. Croft . 2017 The independent influences of heat strain and dehydration upon cognition. Eur. J. Appl. Physiol. 117:1025–1037. 10.1007/s00421-017-3592-2. 28343279

[phy213805-bib-0040] Hew‐Butler, T. , V. Loi , A. Pani , and M. H. Rosner . 2017 Exercise‐associated hyponatremia: 2017 update. Front. Med. 4:21. 10.3389/fmed.2017.00021 PMC5334560 28316971

[phy213805-bib-0041] Hogervorst, E. , W. Riedel , A. Jeukendrup , and J. Jolles . 1996 Cognitive performance after strenuous physical exercise. Percept. Mot. Skills 83:479–488. 8902021 10.2466/pms.1996.83.2.479

[phy213805-bib-0042] Horenstein, C. , M. J. Lowe , K. A. Koenig , and M. D. Phillips . 2009 Comparison of unilateral and bilateral complex finger tapping‐related activation in premotor and primary motor cortex. Hum. Brain Mapp. 30:1397–1412. 18537112 10.1002/hbm.20610 PMC6871138

[phy213805-bib-0043] IOM . 2004 Dietary reference intakes for water, potassium, sodium, chloride, and sulfate. National Academies Press, Washington, DC.

[phy213805-bib-0044] Jenkinson, M. , P. Bannister , M. Brady , and S. Smith . 2002 Improved optimization for the robust and accurate linear registration and motion correction of brain images. NeuroImage 17:825–841. 12377157 10.1016/s1053-8119(02)91132-8

[phy213805-bib-0045] Johnstone, E. , C. D. Frith , T. J. Crow , J. Husband , and L. Kreel . 1976 Cerebral ventricular size and cognitive impairment in chronic schizophrenia. Lancet 308:924–926. 10.1016/s0140-6736(76)90890-4 62160

[phy213805-bib-0046] Kempton, M. , U. Ettinger , A. Schmechtig , E. Winter , L. Smith , T. McMorris , et al. 2009 Effects of acute dehydration on brain morphology in healthy humans. Hum. Brain Mapp. 30:291–298. 18064587 10.1002/hbm.20500 PMC6871128

[phy213805-bib-0047] Kempton, M. , U. Ettinger , R. Foster , S. Williams , G. Calvert , A. Hampshire , et al. 2011a Dehydration affects brain structure and function in healthy adolescents. Hum. Brain Mapp. 32:71–79. 20336685 10.1002/hbm.20999 PMC6869970

[phy213805-bib-0048] Kempton, M. , T. Underwood , S. Brunton , F. Stylios , A. Schmechtig , U. Ettinger , et al. 2011b A comprehensive testing protocol for MRI neuroanatomical segmentation techniques: evaluation of a novel lateral ventricle segmentation method. NeuroImage 58:1051–1059. 21835253 10.1016/j.neuroimage.2011.06.080 PMC3551263

[phy213805-bib-0049] Kenefick, R. W. 2018 Drinking strategies: planned drinking versus drinking to thirst. Sports Med. 48:31–37. 29368181 10.1007/s40279-017-0844-6 PMC5790864

[phy213805-bib-0050] Lindseth, P. D. , G. N. Lindseth , T. V. Petros , W. C. Jensen , and J. Caspers . 2013 Effects of hydration on cognitive function of pilots. Mil. Med. 178:792–798. 23820354 10.7205/MILMED-D-13-00013

[phy213805-bib-0051] Melin, B. , M. Curé , J. M. Pequignot , and J. Bittel . 1988 Body temperature and plasma prolactin and norepinephrine relationships during exercise in a warm environment: effect of dehydration. Eur. J. Appl. Physiol. 58:146–151. 10.1007/BF00636618 3203660

[phy213805-bib-0052] Meyers, S. M. , R. Tam , J. S. Lee , S. H. Kolind , I. M. Vavasour , E. Mackie , et al. 2016 Does hydration status affect MRI measures of brain volume or water content? J. Magn. Reson. Imaging 44:296–304. 10.1002/jmri.25168. 26825048

[phy213805-bib-0053] Montain, S. J. , M. N. Sawka , and C. B. Wenger . 2001 Hyponatremia associated with exercise: risk factors and pathogenesis. Exerc. Sport Sci. Rev. 29:113–117. 11474958 10.1097/00003677-200107000-00005

[phy213805-bib-0054] Morley, J. , G. Beauchamp , J. Suyama , F. X. Guyette , S. E. Reis , C. W. Callaway , et al. 2012 Cognitive function following treadmill exercise in thermal protective clothing. Eur. J. Appl. Physiol. 112:1733–1740. 21892644 10.1007/s00421-011-2144-4

[phy213805-bib-0055] Nakamura, K. , R. A. Brown , D. Araujo , S. Narayanan , and D. L. Arnold . 2014 Correlation between brain volume change and T2 relaxation time induced by dehydration and rehydration: implications for monitoring atrophy in clinical studies. Neuroimage‐Clin. 6:166–170. 25379428 10.1016/j.nicl.2014.08.014 PMC4215533

[phy213805-bib-0056] Nose, H. , T. Morimoto , and K. Ogura . 1983 Distribution of water losses among fluid compartments of tissues under thermal dehydration in the rat. Jpn. J. Physiol. 33:1019–1029. 6674653 10.2170/jjphysiol.33.1019

[phy213805-bib-0057] Pape, L. , and R. Katzman . 1970 Effects of hydration on blood and cerebrospinal fluid osmolalities. Exp. Biol. Med. 134:430–433. 10.3181/00379727-134-34806 5419132

[phy213805-bib-0058] Piil, J. F. , J. Lundbye‐Jensen , S. J. Trangmar , and L. Nybo . 2017 Performance in complex motor tasks deteriorates in hyperthermic humans. Temperature 4:420–428. 10.1080/23328940.2017.1368877 PMC5800368 29435481

[phy213805-bib-0059] Pletzer, B. , M. Kronbichler , M. Aichhorn , J. Bergmann , G. Ladurner , and H. H. Kerschbaum . 2010 Menstrual cycle and hormonal contraceptive use modulate human brain structure. Brain Res. 1348:55–62. 20550945 10.1016/j.brainres.2010.06.019

[phy213805-bib-0060] Popova, N. K. , L. N. Ivanova , T. G. Amstislavskaya , N. N. Melidi , K. S. Naumenko , L. N. Maslova , et al. 2001 Brain serotonin metabolism during water deprivation and hydration in rats. Neurosci. Behav. Physiol. 31:327–332. 11430579 10.1023/a:1010346904526

[phy213805-bib-0061] Rorden, C. , H.‐O. Karnath , and L. Bonilha . 2007 Improving lesion‐symptom mapping. J. Cogn. Neurosci. 19:1081–1088. 17583985 10.1162/jocn.2007.19.7.1081

[phy213805-bib-0062] Sawka, M. N. , A. J. Young , R. P. Francesconi , S. R. Muza , and K. B. Pandolf . 1985 Thermoregulatory and blood responses during exercise at graded hypohydration levels. J. Appl. Physiol. 1985:1394–1401. 10.1152/jappl.1985.59.5.1394 4066570

[phy213805-bib-0063] Serwah, N. , and F. E. Marino . 2006 The combined effects of hydration and exercise heat stress on choice reaction time. J. Sci. Med. Sport 9:157–164. 16621710 10.1016/j.jsams.2006.03.006

[phy213805-bib-0064] Sharma, V. M. , K. Sridharan , G. Pichan , and M. R. Panwar . 1986 Influence of heat‐stress induced dehydration on mental functions. Ergonomics 29:791–799. 3743537 10.1080/00140138608968315

[phy213805-bib-0065] Smith, S. M. 2002 Fast robust automated brain extraction. Hum. Brain Mapp. 17:143–155. 12391568 10.1002/hbm.10062 PMC6871816

[phy213805-bib-0066] Smith, S. M. , M. Jenkinson , M. W. Woolrich , C. F. Beckmann , T. E. J. Behrens , H. Johansen‐Berg , et al. 2004 Advances in functional and structural MR image analysis and implementation as FSL. NeuroImage 23(Supplement 1):S208–S219. 15501092 10.1016/j.neuroimage.2004.07.051

[phy213805-bib-0067] Smith, M. F. , A. J. Newell , and M. R. Baker . 2012 Effect of acute mild dehydration on cognitive‐motor performance in golf. J. Strength Cond. Res. 26:3075–3080. 22190159 10.1519/JSC.0b013e318245bea7

[phy213805-bib-0068] Streitburger, D. P. , H. E. Moller , M. Tittgemeyer , M. Hund‐Georgiadis , M. L. Schroeter , and K. Mueller . 2012 Investigating structural brain changes of dehydration using voxel‐based morphometry. PLoS ONE 7:e44195. 22952926 10.1371/journal.pone.0044195 PMC3430653

[phy213805-bib-0069] Sturm, W. , and K. Willmes . 2001 On the functional neuroanatomy of intrinsic and phasic alertness. NeuroImage 14:S76–S84. 11373136 10.1006/nimg.2001.0839

[phy213805-bib-0070] Szczepańska‐Sadowska, E. , C. Simon‐Oppermann , D. A. Gray , and E. Simon . 1984 Plasma and cerebrospinal fluid vasopressin and osmolality in relation to thirst. Pflüg Arch. 400:294–299. 10.1007/BF00581562 6728650

[phy213805-bib-0071] Szinnai, G. , H. Schachinger , M. J. Arnaud , L. Linder , and U. Keller . 2005 Effect of water deprivation on cognitive‐motor performance in healthy men and women. Am. J. Physiol. Regul. Integr. Comp. Physiol. 289:R275–R280. 15845879 10.1152/ajpregu.00501.2004

[phy213805-bib-0072] Tankus, A. , and I. Fried . 2012 Visuomotor coordination and motor representation by human temporal lobe neurons. J. Cogn. Neurosci. 24:600–610. 22066588 10.1162/jocn_a_00160

[phy213805-bib-0073] Tomporowski, P. D. , K. Beasman , M. S. Ganio , and K. Cureton . 2007 Effects of dehydration and fluid ingestion on cognition. Int. J. Sports Med. 28:891–896. 17907076 10.1055/s-2007-965004

[phy213805-bib-0074] Watson, P. , K. Head , A. Pitiot , P. Morris , and R. J. Maughan . 2010 Effect of exercise and heat‐induced hypohydration on brain volume. Med. Sci. Sports Exerc. 42:2197–2204. 20421835 10.1249/MSS.0b013e3181e39788

[phy213805-bib-0075] Watson, P. , A. Whale , S. A. Mears , L. A. Reyner , and R. J. Maughan . 2015 Mild hypohydration increases the frequency of driver errors during a prolonged, monotonous driving task. Physiol. Behav. 147:313–318. 25890276 10.1016/j.physbeh.2015.04.028

[phy213805-bib-0076] Wittbrodt, M. T. , and M. Millard‐Stafford . 2018 Dehydration impairs cognitive performance: a meta‐analysis. Med. Sci. Sports Exerc. 10.1249/MSS.0000000000001682 29933347

[phy213805-bib-0077] Wittbrodt, M. T. , S. Espinoza , and M. L. Millard‐Stafford . 2015a Biological variation of plasma osmolality obtained with capillary versus venous blood. Clin. Chem. Lab. Med. 53:1613–1619. 25720122 10.1515/cclm-2014-1006

[phy213805-bib-0078] Wittbrodt, M. T. , M. Millard‐Stafford , R. A. Sherman , and C. C. Cheatham . 2015b Fluid replacement attenuates physiological strain resulting from mild hypohydration without impacting cognitive performance. Int J Sport Nutr Exerc. Metab. 25:439–447. 25811390 10.1123/ijsnem.2014-0173

[phy213805-bib-0079] Woolrich, M. W. , B. D. Ripley , M. Brady , and S. M. Smith . 2001 Temporal autocorrelation in univariate linear modeling of FMRI data. NeuroImage 14:1370–1386. 11707093 10.1006/nimg.2001.0931

[phy213805-bib-0080] Worsley, K. J. , A. C. Evans , S. Marrett , and P. Neelin . 1992 A three‐dimensional statistical analysis for CBF activation studies in human brain. J. Cereb. Blood Flow Metab. 12:900–918. 1400644 10.1038/jcbfm.1992.127

